# Characterizations of Six Pomegranate (*Punica granatum* L.) Varieties of Global Commercial Interest in Morocco: Pomological, Organoleptic, Chemical and Biochemical Studies

**DOI:** 10.3390/molecules27123847

**Published:** 2022-06-15

**Authors:** Sara El Moujahed, Rodica-Mihaela Dinica, Mihaela Cudalbeanu, Sorin Marius Avramescu, Iman Msegued Ayam, Fouad Ouazzani Chahdi, Youssef Kandri Rodi, Faouzi Errachidi

**Affiliations:** 1 Laboratory of Applied Organic Chemistry, Faculty of Sciences and Technologies, Sidi Mohamed Ben Abdellah University, Imouzzer Street, B.P. 2202, Fez 30050, Morocco; fouad.ouazzanichahdi@gmail.com (F.O.C.); youssef_kandri_rodi@yahoo.fr (Y.K.R.); 2 Laboratory of Organic Chemistry, Faculty of Sciences and Environment, Dunarea de Jos University of Galati, 111 Domneasca Street, 800201 Galati, Romania; 3 Research Center for Environmental Protection and Waste Management (PROTMED), University of Bucharest, 91–95 Splaiul Independentei, 050095 Bucharest, Romania; mihaelacudalbeanu@gmail.com (M.C.); sorin_avramescu@yahoo.com (S.M.A.); 4 Department of Organic Chemistry, Biochemistry and Catalysis, Faculty of Chemistry, University of Bucharest, 90–92 Soseaua Panduri, 050663 Bucharest, Romania; 5 Laboratory of Functional Ecology and Engineering Environment, Faculty of Sciences and Technologies, Sidi Mohamed Ben Abdellah University, Imouzzer Street, B.P. 2202, Fez 30050, Morocco; iman.mseguedayam@usmba.ac.ma (I.M.A.); errachidifaouzi@yahoo.fr (F.E.)

**Keywords:** *Punica granatum* L., fruit characteristics, juice quality, rinds valorization, phenolic content, antioxidant activity, catechin, high molecular weight

## Abstract

Pomegranate variety properties are important not only to demonstrate their diversity but also to satisfy the current market need for high-quality fruits. This study aims to characterize pomological and physico-chemical features as well as the antioxidant capacity of Moroccan local cultivars (Djeibi, Mersi, Sefri 1 and Sefri 2) compared to the imported ones (Mollar de Elche and Hicaz). The pomological characteristics of varieties were relatively diverse. The juice varieties (PJ) displayed a marketed variability in organoleptic and quality properties, such as the flavor, juice yield, and micro/macronutrients contents. Interrelationships among the analyzed properties and PJ varieties were investigated by principal component analysis (PCA). Dimension of the data set was reduced to two components by PCA accounting for 64.53% of the variability observed. The rinds varieties (PR) were studied for their total phenolics, flavonoids, and condensed tannins quantifications. PR varieties extracts exhibited different levels of free radical scavenging activity and local varieties revealed a greater potential with stability over time. The HPLC-DAD analyses of PR extracts revealed (+) catechin as the major compound, where the highest content was found for the local varieties. The SEC analysis showed the molecular weight distribution of phenolic compounds with a high size of condensed tannins formed by the polymerization of the catechin monomer. Given these properties, this research provides an easy selection of high-quality fruits as potential candidates for local market needs.

## 1. Introduction

Pomegranate (*Punica granatum* L.) is an old-world fruit that enjoys popularity to this day. It grows best in areas that have long, hot, somewhat dry summers and cold winters. The most important growing areas of the world are the Middle East, India, Central Asia, North Africa, and southern Europe [1]. Pomegranate is believed to be originated from Southwest Asia, probably from Iran and some adjoining countries [2]. This fruit is available in many varieties, up to more than 1000 extending throughout the world [3]. Morocco has become one of the world’s leading pomegranate producers where its production had an upward trend during the last years. Indeed, pomegranate production reached a volume close to 133,000 tons due to the Green Morocco Plan (GMP) adopted by the government to improve pomegranate valorization [1]. Half of this production remains in central Morocco on the planes of the Middle Atlas (Beni Mellal-Khenifra area) where there are many pomegranate varieties mainly represented by the Sefri, Djeibi, and Mersi varieties. Until today, researchers have studied around ten Moroccan varieties, while the same genotype is known by different names in different regions [4,5].

Despite its production evolution, pomegranate is less valued in Morocco when compared to other producing countries. The processing and by-product exploitation are still at a minimum level. [6].

Pomegranate offers health benefits to the food system since it contains several classes of compounds that help to prevent diseases such as cancer [7] HIV [8], inflammatory [9] cardiac diseases [10], and some sperm disorders [11]. These benefits are attributed generally to several valuable bioactive compounds such as polyphenols, sugar-containing polyphenolic tannins, and anthocyanins [12]. The edible parts of the fruit contain a considerable amount of sugars, vitamins, polysaccharides, polyphenols, and minerals [13,14]. The inedible parts or rinds represent 50% of total fruit weight and are a potential source of bioactive compounds, mainly punicalagin [15], generated in large amounts as a by-product that is usually poorly exploited [1].

To the best of our knowledge and even though pomegranates are grown in various parts of Morocco, some juice processing industries are still importing foreign varieties for different uses [16]. In this study, the potential of juices and rinds of six pomegranate varieties commonly sold and consumed in Moroccan markets was investigated, where four local (Djeibi, Mersi, Sefri 1, and Sefri 2) and two most known imported (Mollar de Elche and Hicaz) varieties for industrials were studied for well understanding their nutraceutical and molecular diversity. In this context, the aim of this study is to compare the organoleptic and quality properties, as well as the level of bioactive compounds and antioxidant activities between selected varieties (edible and inedible parts), so as to promote local cultivars and provide supporting data in their selection for Moroccan commercial production and industrial processing. Additional objectives in this research are: (i) to study the variations within the local and imported varieties using statistical analyses methods; (ii) to valorize the pomegranate inedible part and investigate the relationships between its total phenolics, tannins, flavonoids contents, and levels of DPPH^•^ scavenging activity, ABTS^•+^ scavenging activity, and total antioxidant capacity; (iii) to characterize the inedible part of varieties for their phenolic compositions and molecular size distributions by chromatographic analysis (HPLC-DAD and SEC).

## 2. Results and Discussion

### 2.1. Pomegranate Varieties Morpho-Pomological and Organoleptic Characteristics

**Figure 1:**
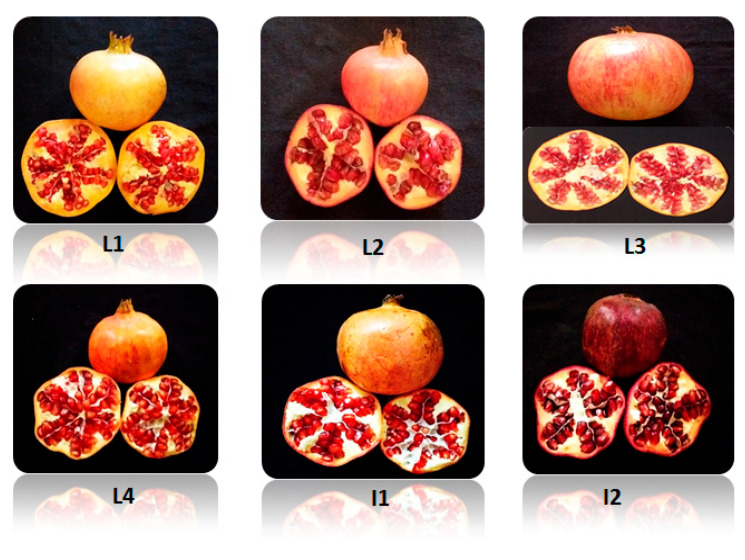
“Djeibi” (L1), “Mersi” (L2), “Sefri 1” (L3), “Sefri 2” (L4), “Mollar de Elche” (I1), and “Hicaz” (I2) pomegranate varieties.

The physical characteristics of the six pomegranate varieties (Figure 1) analyzed are described in Table 1, Table 2 and Table 3. Significant differences (*p* < 0.05) were detected in all measured parameters of the fruit. Fruit weights vary substantially between cultivars L1, L2, L3, L4, I1, and I2, from a minimum of 139.40 g (L2) to a maximum of 479.46 g (L3), as shown in Table 1. Morphometric parameters (length, width, diameter, and weight) showed significant differences between fruit varieties, except L3 and I1, where no differences were observed, representing the highest fruit lengths (7.13 and 7.22 cm), widths (8.50 and 8.31 cm), diameters (8.98 and 8.67 cm), and weights (479.46 and 447.26 g), respectively. However, L4 presented the highest juice and seed contents (67.11 and 4.55%, respectively) and I1 presented superior rinds properties (rind content and rind thickness) with values of 45.27% and 5.46 mm, respectively. No significant differences were detected between L1, L2, and I1 for Rind content %. The lowest values were measured for L2 for the majority of measured parameters. Table 2 represents the arils and seeds physical characteristics determined, resulted in significant differences, especially for aril weights. L3 showed the highest values for aril and seed weights (302.16 and 15.33 g, respectively), which is a determinant parameter of the qualitative aspects of this fruit variety. On the contrary, L2 showed the lowest values for all characteristics, in particular the seed/aril ratio (Table 2), which is an important parameter for pomegranate consumption. Indeed, this parameter is varying between genotypes, where it is often in the 5–10% range [17]. Although less important than seed hardness (Table 3), this characteristic offers a decent indication of aril flavor and palatability since it corresponds to the amount of woody material compared to the total seeds weight, and consumers prefer chewing arils when the woody section is very restricted. Regarding this parameter, no significant difference was shown for L3, I1, and I2.

The following sensory analyses were evaluated in Table 3: Seediness, fruit flavor, and color. Indeed, the latter has a significant impact on the fruit’s eating quality. Fruit color was determined as orange-red for L2- I1, yellow-orange for L3-L4, yellow for L1, and dark red for the imported variety I2. Having red or yellow peels can be used as a potential source for cosmetic colorants or for the textile industry [1]. The red and yellow colors are due to tannins and anthocyanins, which are known also for their significant antioxidant activity. The following varieties: L2, L3, I1, and I2 are soft and semi-seeded, which could be valorized particularly for fresh consumption. Taking into consideration this property, L1 and L4 could be destined for processing.

The results obtained in this research demonstrated that the physical properties of four local cultivars are significantly different in all measured parameters. The imported varieties are not significantly different only for the seed weight/aril weight ratio. For the food and beverage industry, the variety that seems to be most valued has a higher proportion of aril and juice and a lower skin percentage. For those applications, the local variety “Sefri 2” L4 appears to be the most promising with a high percentage of juice (67%) and low skin percentage (28%). However, other varieties L1, L2, and I1 could be also promising for their high rind content (43%, 44%, and 45%), which may be useful, especially in environmental processes [18]. The variety “Sefri1” (L3) with its bigger fruits might involve cultivars development with higher agronomic potential. Varieties carrying high seed contents might be valorized for oil extraction that can be used in formulations of novel foods and cosmetics [6].

### 2.2. Physico-Chemical Characteristics of Pomegranate Juice (PJ) Varieties

The results for total sugars (TS), total proteins (TP), pH, titratable acidity (TA), total soluble solids (TSS), and maturity index (MI) from the different PJ varieties are presented in Table 4. Significant differences (*p* < 0.05) were revealed among the pomegranate varieties for all parameters except total sugars (TS).

As shown in Table 4, the concentration of TS was similar for all studied varieties, with no significant difference (*p* > 0.05). TS concentration was between 11.17 (L3) and 13.18 (I1) g/100 mL. Previous studies reported high TS values of Moroccan PJ, varying from 16.1 g/100 g for “Mersi” to 19.3 g/100 g for “Sefri” where the main predominant sugars were fructose and glucose [19]. The result obtained for I1 is confirmed by Melgarejo et al. [20], who reported that the average of total sugars for sweet Spanish PJ is 13.50 g/100 g of juice. I2 has shown a low content of total sugars when compared to previous studies on Turkish PJ [21]. TP contents presented low values ranging from 0.66 to 1.16 g/100 mL. TP content in PJ has rarely been reported in previous studies and Erkan-Koç et al. [22] confirmed that protein content in pomegranates is very low. The pH values ranged between 3.67 (L1) and 4.56 (I2). The pH value obtained for I2 is higher than the values reported by Cam et al. [12] on pomegranate cultivars grown in Turkey, while the pH value obtained for I1 is similar to the values reported on Spanish varieties by Martinez et al. [23]. The TA content ranged between 2.6 (L3)–5.5 (I2) g of citric acid/L. For Moroccan “Sefri” varieties (L3, L4), similar results were reported by Hmid et al. [5]. Likewise, previous studies confirmed that Moroccan local varieties TA ranged from 2.4 to 4.8 g/L [19]. However, our TA values for I1 were higher than values reported previously for this variety [23]. The highest and the lowest TSS contents were recorded by imported varieties, with values of 29.2 °Brix for Spanish PJ and 12.8 °Brix for Turkish PJ, while these results were higher than values previously reported (12–14 °Brix) for Mollar de Elche PJ [23] and also for Moroccan local varieties [5]. Finally, some authors adopted the maturity index (TSS/TA) to classify pomegranate varieties which is responsible for pomegranate taste and flavor [23,24]. The following classification has been established for Spanish varieties: maturity index (MI) = 5–7 for sour, MI = 17–24 for sour-sweet, and MI = 31–98 for sweet varieties. Taking into consideration this simple rule, it can be concluded that all studied varieties are sweet except the Turkish variety (I2), which is sour-sweet. According to the results, origin of variety has a significant impact on such parameters (TSS, pH, and TA). Because of their high maturity index (MI), the varieties studied were appropriate either for direct consumption or industrial juice processing.

The results for ascorbic acid (AA), total phenolic compounds (TPC), total flavonoids (TF), total tannins (TT), hydrolyzable tannins (HT), and total carotenoids (TCA) contents from the different PJ varieties are presented in Table 5. Highly significant differences (*p* < 0.01) were revealed among the pomegranate varieties for the majority of measured contents.

Among the six PJ varieties, there was a considerable difference in TF, TT, HT, and TCA contents. High AA content was recorded by the local variety L2 with a value of 851.56 g AAE/L of PJ, while there was no significant difference between imported varieties. The data obtained in this study are higher than the values reported in the literature [25,26]. Despite its challenging instability [25,27] and either alone or in combination with phenolics, the AA remained responsible for the antioxidant activity of pomegranate [28]. TPC for local varieties ranged from 885 mg to 1216 mg GAE/L, I1 and I2 had contents of 995 mg and 1348 mg GAE/L, respectively. I2, L1, and L4 registered the highest contents of TPC. A significant variation in TF, TT, HT, and TCA concentrations was found where the high values were registered by imported varieties (I1, I2). The Turkish variety I2 had the highest contents of TF and HT with values of 140.44 mg QE/100 mL and 362.51 mg TAE/100 mL, respectively. Çam et al. showed that the TPC of eight pomegranate varieties extensively cultivated in Turkey varied between 2083 and 3436 mg/L [29], and our data for I2 were lower than their results. The I1 variety registered the highest contents in TT with a value of 1456.14 mg/100 mL. The reported levels of total phenolics in the literature were between 2674 and 4210 mg GAE/L for Spanish varieties [30], 144 and 10,086 mg GAE/L for Turkish varieties [31], and 410.1 and 834.3 mg GAE/kg for Moroccan varieties [19]. These differences in phenolics contents may be impacted by variations in phytochemical content, variety, extraction procedure, and environmental conditions. The I1 variety contains a high TCA content with a value of 1.87 mg β-CAE/100 mL. β-carotene is the main carotenoid found in pomegranate making up 93% of the total carotenoids content [32]. A few studies reported results about total carotenoids content in pomegranate. Goula et al. were among the first experimenters of carotenoids extraction from pomegranate peels using vegetable oils, where the yield obtained was around 0.52 mg β-carotene/100 g of dry matter [32]. The pigmentation level of pomegranate juice is closely linked to carotenoids and anthocyanins, where they work like a balance: a reduction in carotenoids imparts a yellow color, an increase in anthocyanins mainly gives the red color of the fruit [33]. In regard to the chemical composition, the data obtained are very interesting, based on the bioactivity of pomegranate tannins enhancing human health; as they could be absorbed by the intestine as proved by Filippich et al. [34].

### 2.3. Principal Component Analysis (PCA)

Grouping of pomegranate varieties (L1, L2, L3, L4, I1, and I2) using PCA was based mainly on the first two PC that account for 64.53% of the variability observed, i.e., for 38.17% and 26.36%, respectively (Table 6). The most important physical variables integrated by PC1 were fruit length (L), width (W), diameter (D), weight (Wg), and rind thickness (Rth), while the physico-chemical variables were total soluble solids (TSS), maturity index (MI), and total tannins (TT).

**Figure 2:**
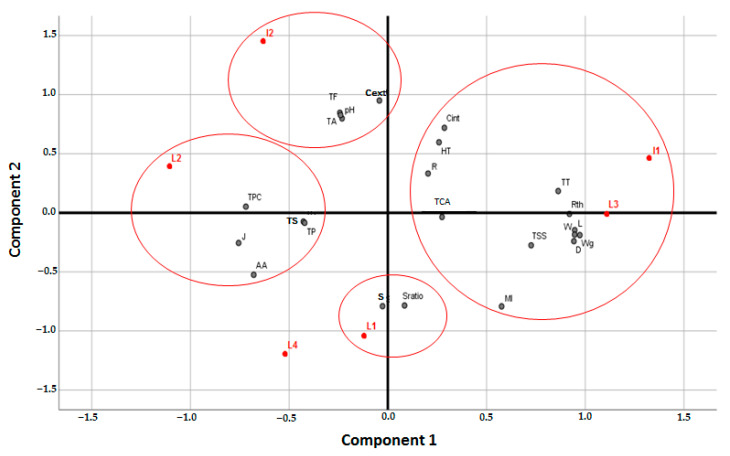
Principal component analysis (PCA) of selected varieties (L1, L2, L3, L4, I1, and I2) using fruit morphology and juice physico-chemical characters.

The correlation between PC1 and fruit external color (Cext) was negative and, relatively, less important for internal color (C int) (Table 6). PC1 separated varieties having large and/or less colored fruits as I1 and L3 from those with small and/or more colored ones as L1, L2, L4, and I2. PC2 is correlated mainly to juice characteristics. It is correlated positively with juice color (C int), pH, total acidity (TA), total flavonoids (TF), and hydrolysable tannins (HT), and negatively with juice% (J), seed% (S), seed ratio (S ratio), maturity index (MI), and ascorbic acid content (AA) (Table 6). It was possible to differentiate, according to PC2, varieties with high acidity and/or less juice content and intense color (I1, I2) from others with low acidity and/or less color with a high juice content (L1, L4). The finding allowed a specific differentiation pattern in which five groups are distinguished: I3-I1, I2, L2, L1, and L4 (Figure 2). Indeed, the variety L1 contributes to the negative part of PC2 and is correlated with the S and S ratio. The two varieties I1 and L3 contribute to the positive part of PC1 and are correlated with L, W, D, W, Rth, R, TSS, TCA, TT, and MI. The variety I2 contributes more to the positive part of PC2 and correlates with TF, TA pH Cext. The L2 variety contributes massively to the negative part of PC2 and is correlated with the parameters TPC, TS, TP, J, and AA (Figure 2). In contrast, the L4 variety was not correlated with morphological and biochemical traits studied. In light of these findings and the previously stated observations, it was discovered that there is a significant difference between the varieties which might be a useful tool for quickly analyzing technological characteristics, such as fruit shape and weight, phenolics content, and juice quality, of pomegranate varieties with commercial interest.

### 2.4. Pomegranate Rinds (PR) Varieties Chemical Characterizations

The most common by-product of the agri-food pomegranate processing chain is the pomegranate rind (PR). The existence of bioactive compounds appropriate for use in the creation of food additives has received much interest in recent decades to PR. The purpose of this study was to investigate and characterize the nutritional and nutraceutical quality of PR of six pomegranate varieties, which were chosen based on their high commercial interest in the Moroccan market.

#### 2.4.1. Microplate Quantifications in Pomegranate Rinds (PR)

**Figure 3:**
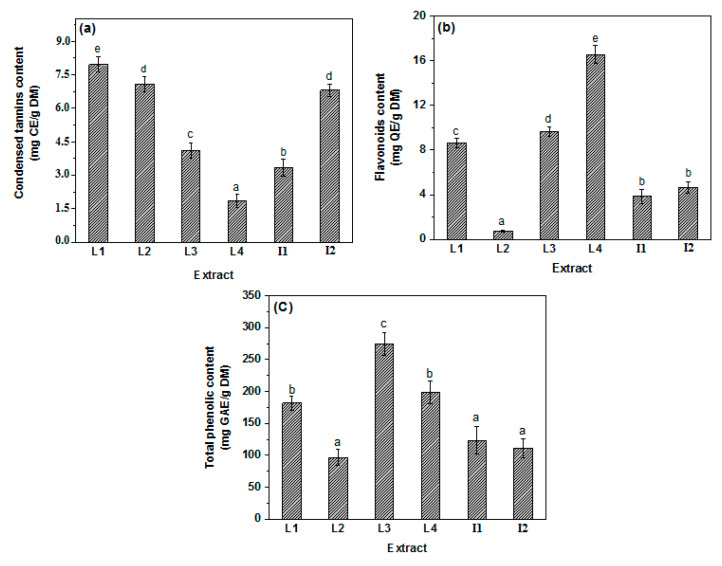
Condensed tannins (mg of catechin equivalent/g of dry matter) (**a**), flavonoids (mg of quercetin equivalent/g of dry matter) (**b**) and total phenolic compounds (mg of gallic acid equivalent/g of dry matter) (**c**) contents; values with different letters a, b, c, d, e for each graph are statistically different (Duncan test, *p* = 0.05).

Figure 3 illustrates total phenolic compounds content (TPC), condensed tannins content (CTC), and flavonoids content (TFC) found in PR extracts. The results of this study show that the variations in phenolics contents of extracts from PR varieties were significant (*p* < 0.05). The highest TPC was found in L3 (277.86 mg GAE/g DM). The TPC for L2, I1, and I2 was comparable, recording the lowest contents ranging up to 101.09, 131.08, and 111.48 mg GAE/g DM, respectively. For the TFC, the L4 recorded the highest content (16.55 mg QE/g DM), while the lowest content was registered for L2 (0.79 mg QE/g DM). However, there is no significant difference between L1 and L3 varieties. For the CTC, L1 showed the highest content of condensed tannins with a value of 7.84 mg CE/g DM. L3 and I1 showed comparable contents with no significant difference. The results provide significant information about phenolic compounds content in different PR varieties, where the Moroccan local ones L1, L2, L3, and L4 showed high contents compared to I1 and I2, the imported ones. Levels of bioactive compounds were previously studied in the peel of Spanish varieties [35]. Rinds extracts had a high total content of polyphenols (ranging from 161 to 240 mg GAE/g), where for the aqueous extract a value of 161 mg GAE/g was registered, which is higher than the result obtained for I1. Rinds extracts from the Hicaz pomegranate cultivar and 3 genotypes from Turkey have been studied [36]. This variety had approximately 5.9-fold higher total phenolics contents than juice extract, where total tannins contents varied between 86.62 and 183.18 μg TAE/mg in peel extract and total flavonoids content of extracts varied between 9.44 and 20.52 μg QE/mg in peel, which are higher than our results. Benchagra et al. [4] confirmed the richness of the Moroccan Sefri variety with TPC and TFC and registered higher values of these contents.

#### 2.4.2. Antioxidant Activities of Pomegranate Rinds (PR) Varieties

**Figure 4:**
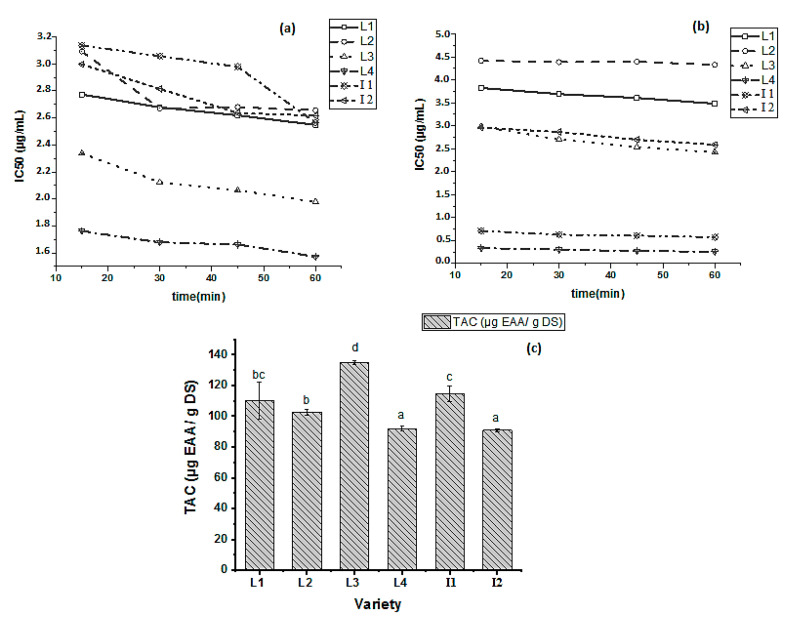
Antioxidant capacity of L1, L2, L3, L4, I1, and I2 varieties by DPPH^•^ method (expressed as IC_50_ μg/mL) (**a**), ABTS^•+^ method (expressed as IC_50_ μg/mL) (**b**), and total antioxidant capacity (TAC) method (expressed as μg eq ascorbic acid/g dry sample) (**c**).

The antioxidant activity of food products can be assessed using a variety of methods. This wide range of approaches is due to the reality that none of them can accurately assess the overall antioxidant potential in a food system. In this study, the in vitro antioxidant activity of pomegranate rind extracts from L1, L2, L3, L4, I1, and I2 varieties was measured by three different analytical methods: ABTS^•+^, DPPH^•^, and TAC (Figure 4). For DPPH^•^ (Figure 4a) and ABTS^•+^ (Figure 4b), the results are expressed as the evolution over time of the half-maximal inhibitory concentration (IC_50_ μg/mL). The total antioxidant capacity (TAC) (Figure 4c) was determined by the phosphate–molybdate method and the results were expressed as μg equivalent of ascorbic acid/g dry sample (μg EAA/g DS). The data presented the mean of three replicates from accession. Duncan test was used to determine statistically significant differences at (*p* < 0.05) as identified by different letters.

High antiradical activity was measured by extract varieties for DPPH^•^ (Figure 4a). The IC_50_ values ranged from 1.76 (L4) to 3.09 (L2) μg/mL, showing a slight decrease in their stability after 15 min of incubation except for L2 and I2 which remained stable over time after 30 min and 45 min of incubation, respectively. The hierarchy for DPPH^•^ antioxidant capacity following the IC_50_ values was L4 > L3 > L1 > I2 > L2 > I1. Indeed, this classification is proportional to total flavonoids contents (TFC) and represents the same hierarchy found in this quantification (Figure 3b). This finding is in accordance with previous studies showing that phenolic compounds significantly boost antiradical activity, because of their optimal chemical structure for capturing free radicals [37].

The ABTS^•+^ method is a good way to determine hydrophilic substances’ antioxidant activity. Because of its solubility in aqueous medium, the ABTS radical cation interacts efficiently with antioxidant substances and provides, additionally to DPPH^•^, a whole picture of radical quenching ability [38]. Pomegranate extracts possessed a high antioxidant capacity for ABTS^•+^ (Figure 4b), with IC_50_ values as low as ranging from 0.71 to 4.41 μg/mL and remaining constant over time (after 60 min) in the case of L2, L4, and I1. Whereas, L1, L3, and I2 showed a high activity with a slight decrease in their stability after 15 min of incubation. The lowest IC_50_ was recorded by L4 (0.71 μg/mL) and the highest was taken by L2 (4.41 μg/mL) at 15 min. The hierarchy for ABTS^•+^ antioxidant capacity following the IC_50_ values was L4 > I1 > I2 > L3 > L1 > L2. Indeed, this classification is inversely proportional to condensed tannins content (CTC), where the more the latter increases the more the ABTS^•+^ antioxidant activity decreases. Previous studies revealed differences highly significant in hydrophilic and lipophilic antioxidant activity across cultivars, which are dependent on a variety of characteristics such as cultivars, maturity index, geographical origin, and so on [39,40].

The total antioxidant capacity method is based on the reduction of molybdate Mo (VI) to molybdenum Mo (V) by antioxidant substances (Figure 4c). Because the antioxidant activity is represented in μg eq ascorbic acid/g of each extract, it has the benefit of being a quantitative approach. The antioxidant potential ranged from 90.95 (I2) to 135.07 (L3) μg EAA/g DS. All extracts showed significant differences (*p* < 0.05) except for L4 and I2. The hierarchy for total antioxidant capacity was L3 > I1 > L1 > L2 > L4 > I2. Previous studies demonstrate that the antioxidant activity by the phospho–molybdenum method revealed the pomegranate methanolic extract as a potential source of antioxidant compounds responsible for the demonstrated activity with values going up to 5067μmol ascorbic acid/g extract at a concentration of 80 μg/mL [41]. Karray et al. [37] proved that pomegranate peels have a high TAC when compared to Spirulina and Moringa. Alcade et al. [42] evaluated the antioxidant capacity of phenolic compounds according to their chemical structures and reported weak correlations between different tests provided and a unique behavior of each compound as a response to each method used. Nonetheless, It is worth noting that the difference resulting in TAC hierarchy compared to other radical scavenging activities might be dependent on other synergetic or antagonistic interfering interactions other than phenolics. Indeed, previous studies reported that ascorbic acid can produce synergy or antagonism in TAC assays. Likewise, sugars such as glucose, sucrose, and fructose contributed also to this reaction and played a determining role in TAC [43]. Taken together, slight compositional variations can influence TAC values through intricate interactions among compounds with and without strong antioxidant effects.

The results obtained confirmed the importance of PR as a natural antioxidant. In summary, the DPPH^•^, ABTS^•+^, and TAC measurements indicate the stability of bioactive substances from PR extracts, meaning that the antioxidant activities remain constant and can act over time.

Pearson’s correlation coefficients between the means of phenolics contents (TPC, TFC, and CTC) and antioxidant activities (DPPH^•^, ABTS^•+^, and TAC) were computed and reported in Table 7. A statistically highly significant correlation (*p* < 0.01) coefficient was found between TFC and IC_50_ (DPPH^•^) (r^2^ = −0.954), and a significant correlation (*p* < 0.05) between TPC and IC_50_ (DPPH^•^) (r^2^ = −0.724), CTC, and IC_50_ (ABTS^•+^) (r^2^ = 0.895). Correlation coefficients were high among two antioxidant activity values (DPPH^•^ and ABTS^•+^). However, the TAC value was not significantly correlated with any of the variables, except for TPC with a low coefficient (r^2^ = 0.624). The results obtained confirm the high correlation and the hierarchy reported in the section above, between DPPH^•^–TFC and ABTS^•+^–CTC.

#### 2.4.3. Steric Exclusion Chromatography (SEC) Analysis of PR Varieties

**Figure 5:**
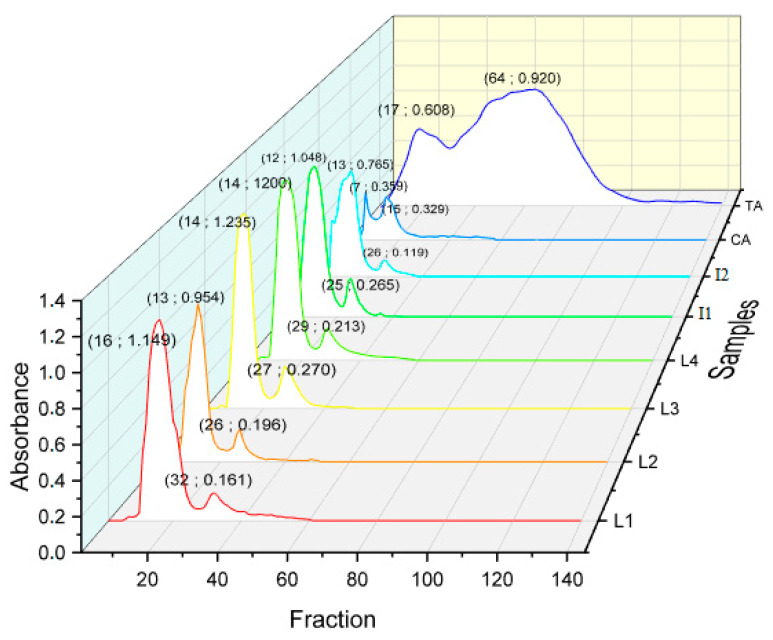
Molecular distribution of pomegranate varieties extracts (L1, L2, L3, L4, I1, and I2) catechin (CA) and tannic acid (TA). Inset: (fraction N°; absorbance).

The presence of tannins as major constituents in phenolics composition of pomegranate led to the analysis of their molecular distribution by gel filtration. Thus, the polymerization profiles of each crude extract from varieties L1, L2, L3, L4, I1, and I2, provide data to understand the molecular sizes of active substances and their usefulness in further applications. Catechin and tannic acid were used as references to follow the polymerization degree of condensed tannins and hydrolyzable tannins, respectively. The elution profiles are illustrated in Figure 5.

Comparing elution profiles in Figure 5, it can be concluded that the different rinds varieties highlight, qualitatively and quantitatively, differences in molecular distributions of tannins content. As reported in our previous work [1], the smaller the fraction number, the highest the molecular weight. The profile contained two regions: the high mass fraction from 5 to 25 and the low mass fraction from 25 to 100, which has been reduced to a single high- and low-intensity peak for each extract. I1 exhibited the highest molecular weight (12; 1.048), followed by I2 (13; 0.765) and L2 (13; 0.954). Medium polymers size is attributed to L1 (16; 1.149), L3 (14; 1.235), and L4 (14; 1.200). The high molecular size peaks match with the elution profile of catechin (15; 0.329), which is the main monomer of condensed tannins polymerization [44]. Indeed, it can be concluded that PR extracts are characterized by the high molecular weight manifested by condensed tannins. Previous studies confirmed that pomegranate peel is characterized by the presence of high molecular weight phenolics, such as ellagitannins and proanthocyanidins [45]. On the other hand, the tannic acid elution profile illustrated a self-polymerization in time showing a slow elution within Sephadex gel beads, translated by the small size of molecules. For that reason, it is worth noting that extracts obtained from L1, L2, L3, L4, I1, and I2 all have high molecular sizes of condensed tannins, which could be valued as potential agents in environmental applications [18].

#### 2.4.4. High-Performance Liquid Chromatography with a Diode-Array Detector (HPLC-DAD) Analysis of PR Varieties

**Figure 6:**
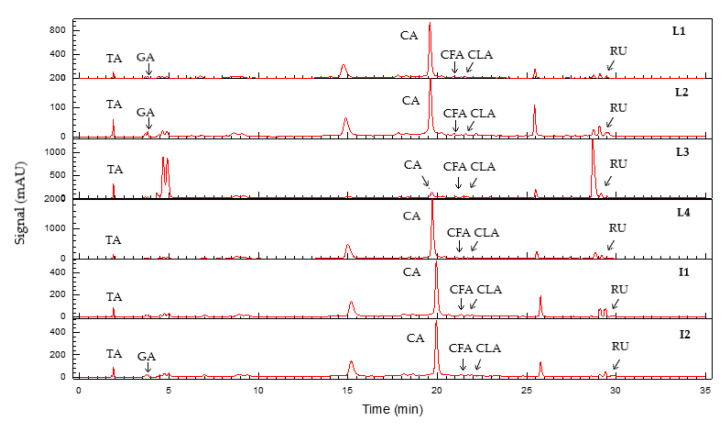
HPLC-DAD chromatograms of L1, L2, L3, L4, I1, I2 PR extracts. TA—tannic acid; GA—gallic acid; CFA—caffeic acid; CLA—chlorogenic acid; CA—catechin; RU—rutin recorded at 250 nm.

The PR phenolic compounds were identified and quantified by HPLC-DAD analysis, as the main compounds of this vegetable biomass [46,47]. From the sixteen standard compounds (tannic acid, gallic acid, (+) catechin, (−) epicatechin, caffeic acid, chlorogenic acid, *p*-coumaric acid, rutin, naringenin, naringin, quercetin, daidzein, genistein, hyperoside, delphinidin, and malvidin) that were proposed for HPLC-DAD analysis, six phenolic compounds were identified (tannic acid, gallic acid, (+) catechin, caffeic acid, chlorogenic acid, and rutin) in the pomegranate extracts (L1, L2, L3, L4, I1, I2). The phenolic standard compounds: (−) epicatechin, *p*-coumaric acid, naringenin, naringin, quercetin, daidzein, genistein, and hyperoside were characterized by the absence of peaks; moreover, the anthocyanins that have absorbance in the range between 400 and 600 nm (λ_max_ = 520 nm) [48] were characterized by the absence of peaks (delphinidin and malvidin recorded at 510 nm). The quantification of PR extracts was conducted at the λ_max_ of each standard compound described in the method. The HPLC-DAD profiles of all PR extracts (L1, L2, L3, L4, I1, I2) are summarized in Table 8, and chromatograms recorded at 250 nm for all PR extracts are shown in Figure 6.

**Figure 7:**
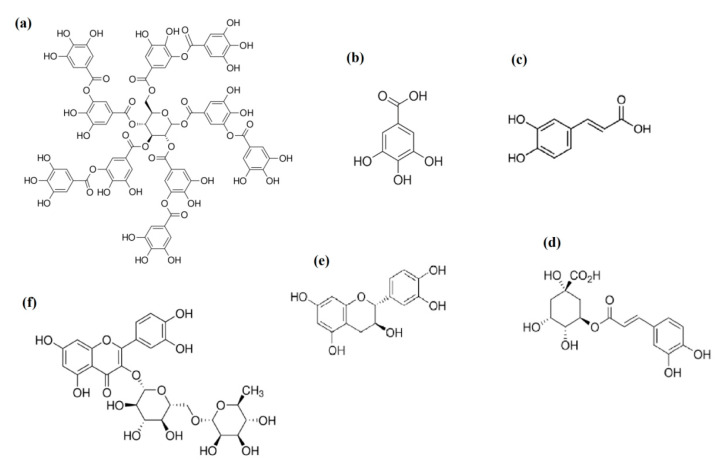
Chemical structures of identified phenolic compounds in PR varieties extracts; (**a**) tannic acid; (**b**) gallic acid; (**c**) caffeic acid; (**d**) chlorogenic acid; (**e**) catechin; (**f**) rutin.

**Figure 8:**
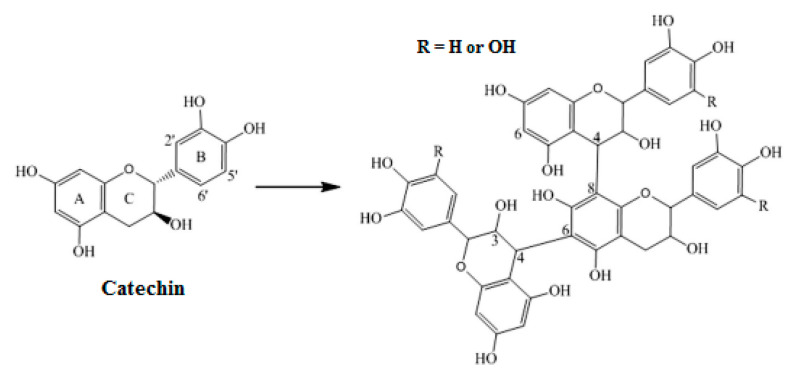
Polymerization of catechin monomer to a general structure of condensed tannins.

As shown, tannic acid, (+) catechin, caffeic acid, chlorogenic acid, and rutin have been identified in all PR extracts (L1, L2, L3, L4, I1, I2). (+) Catechin is the most abundant compound in all varieties of extracts, ranging from 93.63–1579.91 mg/kg dw. This result confirms the finding in Section 2.4.1 of TFC determination and in the section above (SEC), where the molecular peaks match well with the catechin elution profile (Figure 5). The concentration range of other phenolic compounds (tannic acid, gallic acid, caffeic acid, chlorogenic acid, and rutin) is much lower than the principal PR compound (catechin). All varieties of extracts contain phenolic acids, except L3, L4, and I1 in which a lack of gallic acid has been observed. The richest chemical composition was found in L4 followed by L1 local varieties with a total amount of (+) catechin ranging up to 1579.91 and 690.84 mg/kg dw, respectively. The (+) catechin contents in PR are highly dependent on variety (Table 7). Different from our results, lower catechin contents were published for different pomegranate varieties [49,50,51]. Mabrouk et al. [52] have confirmed catechin as the main abundant flavonoid in pomegranate rinds and predominant phenolic compounds were reported as catechin (963.36 mg/kg dw) and gallic acid (278.44 mg/kg dw) Hmid et al. [53] studied phenolic compounds of eighteen pomegranate cultivars in Morocco. Chlorogenic, caffeic, ferulic, gallic, and ellagic acids, as well as catechin, phloridzin, quercetin, epicatechin, and rutin, were identified as phenolic compounds. The difference in identified compounds, when compared to previous studies, confirmed that the extraction procedure used in this work succeeded to select the condensed tannins fraction represented by the high content of (+) catechin. Indeed, catechin contains a flavon-3-ol structure (Figure 7). It has been reported previously as a condensed tannin precursor (Figure 8); these latter are polymerized products of flavanols and they are also referred to as proanthocyanidins [54] The high concentration of catechin contained in our PR varieties (especially the local varieties L1 and L4) confers to this vegetable biomass specific properties in biomaterials stabilization [18,54].

## 3. Materials and Methods

### 3.1. Chemicals and Instruments

Except as otherwise noted, all reagents and compounds were analytical grade and were utilized without additional purification. All solvents (hexane/acetone/ethanol/methanol) were redistilled before use. DNS reagent (3,5-dinitrosalicylic acid), Biuret reagent (cupric sulfate (CuSO_4_.5H_2_O), sodium potassium tartrate (NaKC_4_H_4_O_6_.4H_2_O), sodium hydroxide (NaOH) and potassium iodide (KI), hydrochloric acid (HCl), sulfuric acid (H_2_SO_4_), sodium hydroxide (NaOH), sodium carbonate (Na_2_CO_3_), sodium thiosulfate (Na_2_S_2_O_3_), sodium nitrite (NaNO_2_), lithium chloride (LiCl), and gel filtration for chromatography Sephadex G-50 (superfine beads size) were purchased from Sigma-Aldrich (Casablanca, Morocco). Folin–Ciocalteu reagent, aluminum trichloride (AlCl_3_), DPPH radical (2,2-diphenyl-1-picrylhydrazyl), ABTS radical cation (2,2′-azino-bis (3-ethylbenzothiazoline-6-sulfonic acid)), and TAC reagent (0.6 M sulfuric acid, 28 mM sodium phosphate, and 4 mM ammonium molybdate) were obtained from Sigma-Aldrich (Steinheim, Germany). Standards such as gallic acid, tannic acid, quercetin, (+) catechin, ascorbic acid, D-glucose, β-carotene, and bovine serum albumin (BSA) were from Sigma-Aldrich (Steinheim, Germany).

Total soluble solids were measured using a digital refractometer (A.KRÜSS Optronic GmbH, Germany) and the extracts concentration was performed by a freeze dryer (Christ Alpha 1-4 LD plus (Martin Christ, Germany). The absorbance measurements were performed using an ultraviolet/visible spectrophotometer (Shimadzu UV-1600 PC UV spectrophotometer and a multi-well plate reader (Tecan Pro 200, Tecan Trading AG, Männedorf, Switzerland). Chromatographic separations were performed using an L-3000 high-performance liquid chromatography system (Rigol Technologies, Beijing, China).

### 3.2. Vegetable Material: Varieties Collection

Pomegranate (*Punica granatum* L.) rinds varieties were collected from six varieties with high commercial interest, used by Moroccan agri-food industries in the process of pomegranate juice manufacturing. Four local and two imported varieties (Table 9) were purchased from the imported fruit wholesale market during the month of October 2021 at the same maturity stage. Fifteen fully mature, ripe, and medium-sized pomegranate fruits from each variety were used for the determination of physical properties. Fruits were firstly washed in tap water, followed by distilled water and ultra-pure water. The juices were squeezed by manual wooden press from arils of fruits and seeds were manually separated, dried, and hermetically kept in closed boxes until further use. The juice samples (L1, L2, L3, L4, I1, and I2) were frozen at −25 °C pending the analysis. Rinds were watery washed, disinfected, rinsed with distilled water, and dried in the shade, until weight constancy, away from humidity and light, and at ambient temperature. After drying, rinds were crushed using an automated grinder and kept hermetically in closed containers at 4 °C until achievement of the assay. Three repeats were maintained for each analysis.

### 3.3. Determination of Basic Parameters and Physical Characteristics of Pomegranate Varieties

The physical properties of pomegranate fruits from each variety were examined independently. The peels were physically separated from the fruit after weighting. The juice and peels percentages for each variety were measured. Arils and seeds characteristics [23] and chemical analysis were realized on fifteen pomegranate fruits sampled from each variety. The following pomological properties were measured on the fruits: weight (g), length (cm), width (cm), diameter (cm), rinds (%), juice (%), seeds (%), and skin thickness (mm). The following arils and seeds characteristics were analyzed [17]: aril weight (g), seed weight (g), seed length (mm), seed diameter (mm), and woody portion index measured as the seed weight/aril weight ratio (%). In addition, total soluble solids (TSS) content (°Brix) and pH of juices were also adopted. Brix° value was measured using a digital refractometer (A.KRÜSS Optronic GmbH, Germany). Finally, the hardness of seeds, flavor, and visual color was assessed by a panel of six experienced tasters.

### 3.4. Physico-Chemical Analysis of Pomegranate Juice Varieties (PJ)

The clarified PJ from selected pomegranate varieties were evaluated for their main physico-chemical parameters. PJ samples were analyzed for total titratable acidity (TA) which was determined by titration to pH 8.1 using a standard solution of NaOH (0.1 M), and 5 mL of juice was diluted to 50 mL with distilled water [55]. Titratable acidity was expressed as g citric acid/L of juice and the maturity index (MI) (Brix°/acidity) was calculated. Total sugars (TS) [56] were expressed as g glucose/100 mL of PJ, total proteins (TP) [57] were expressed as g BSA/100 mL of PJ, and ascorbic acid (AA), according to the iodometric titration method [58], was expressed as g ascorbic acid/L of PJ. Total phenolic compounds (TPC) were determined according to the Folin–Ciocalteu method [59] and expressed as g gallic acid equivalent/L of PJ and total flavonoids (TF) were determined with the aluminum trichloride method [60] expressed as mg quercetin equivalent/100 mL of PJ. Total tannins (TT) [61], hydrolyzable tannins (HT) [62], and total carotenoids (TCA) [63] contents were expressed as mg/100 mL of PJ, mg tannic acid equivalent/100 mL of PJ and mg β-carotene equivalent/100 mL of PJ, respectively. Analyses were performed in triplicate and repeated twice. The results were evaluated statistically (Section 3.6.5).

### 3.5. Pomegranate Rinds (PR) Extraction Procedure

Pomegranate rinds (PR) extraction from the varieties (L1, L2, L3, L4, I1, and I2) was performed by aqueous-based solid–liquid extraction in duplicate in a round-bottom flask with stirring (100 rpm) in ultra-pure water. For each rinds variety, a ratio of 1:10 (*w*/*v*) rind powder-to-heated ultrapure water (100 °C) was employed and extraction time was 12 h away from light at 20 °C. Afterwards, extracts obtained were first centrifuged at 5000 rpm for 15 min at 5 °C, the supernatant was vacuum filtered, frozen at −80 °C for 24 h, and finally lyophilized in a freeze dryer (Christ Alpha 1-4 LD plus (Martin Christ, Germany)) for 3 days. Extracts samples (L1, L2, L3, L4, I1, and I2) were then stored at 4 °C for the following studies.

### 3.6. Physico-Chemical Analysis of Pomegranate Rind Varieties (PR)

#### 3.6.1. Microplate Determinations of Phenolic Compounds Contents

Extracts samples from (L1, L2, L3, L4, I1, and I2) were evaluated for their main phenolic compounds by microplate determinations. Total phenolic compounds (TPC) were quantified according to the Folin–Ciocalteu method, as described by Kruawan and Kangsadalampai [64] and the results were expressed as gallic acid equivalent (GAE) per gram of dried matter (mg GAE/g DM). The aluminum chloride colorimetric method was used to determine the total flavonoids content (TFC) following the procedure employed by Busuioc et al. [65]. Results were expressed as quercetin equivalent (QE) per gram of dried matter (mg QE/g DM). The vanillin–HCl methodology adapted by Cudalbeanu et al. [66] was used to determine the condensed tannins contents in aqueous extracts of pomegranate varieties. The results were expressed as catechin equivalent (CE) per gram of dried matter (mg CE/g DM). The absorbance was measured by a 96-well plate analysis using a microplate reader (Tecan Pro 200, Tecan Trading AG, Männedorf, Switzerland). All measurements were carried out in triplicate.

#### 3.6.2. Antioxidant Activity of Pomegranate Rind Varieties (PR)

- 2,2-diphenyl-1-picrylhydrazyl free radical-scavenging assay (DPPH^•^)

Extracts from PR varieties were prepared in concentrations ranging from 125 mg/mL to 0.97 μg/mL using successive dilution (1/2) in a 96-well microplate. Briefly, 100 µL of 100 µg/mL 2,2-diphenyl-1-picrylhydrazyl (DPPH^•^) solution was added to 100 µL of extract. Samples absorbance was measured at 517 nm using a multi-well plate reader (Tecan Pro 200) after 15, 30, 45, and 60 min of incubation at room temperature [66]. The control samples used were the DPPH^•^ solution mixed with ultrapure water. The DPPH^•^ inhibition percentage was calculated using the following formula, and the IC_50_ values were determined graphically as the concentration reducing 50% of DPPH^•^ activity at each reading time.

$$\%\mathrm{Inhibition}=\frac{\mathrm{Acontrol}\;-\;\mathrm{Asample}\;}{\mathrm{Acontrol}\;}\times 100$$

where A_control_ is the absorbance of the control reaction and A_sample_ is the absorbance of samples. The control was performed in the same method, except that ultra-pure water was used instead of sample. The test was carried out in triplicate.

- 2,2′-azino-bis(3-ethylbenzothiazoline-6-sulfonic acid) free radical-scavenging assay (ABTS^•+^)

Extracts from PR varieties were prepared in concentrations ranging from 125 mg/mL to 0.97 μg/mL using successive dilution (1/2) in a 96-well microplate. Briefly, 100 µL of 2,2′-azino-bis(3-ethylbenzothiazoline-6-sulfonic acid) (ABTS^•+^) solution in methanol (1:60) was mixed with 100 µL of extract. The absorbance of the samples was recorded at 734 nm using a Tecan Pro 200 multi-well plate reader after 15, 30, 45, and 60 min of incubation at room temperature. For control samples, the extract was replaced with ultrapure water [67]. The ABTS^•+^ inhibition percentage was calculated using the same formula of the DPPH^•^ assay (section above) and the IC_50_ values were determined graphically as the concentration reducing 50% of ABTS^•+^ activity at each reading time.

- Total antioxidant capacity (TAC)

The total antioxidant capacity was determined as described by Prieto et al. [68]. The total antioxidant activity of the PR varieties was determined by the phosphate–molybdate method. The test is based on the reduction of Mo (VI) to Mo (V) by the extract and green phosphate/Mo (V) complex in the acidic medium. An aliquot of 0.1 mL of sample solution containing a reducing species (in water) was mixed with 1 mL of reagent solution (0.6 M sulfuric acid, 28 mM sodium phosphate, and 4 mM ammonium molybdate). Extracts were prepared at a concentration of 90 µg/mL using ultra-pure water as a solvent. Then, samples were mixed with the reagent solution and incubated in a thermal block at 95 °C for 90 min. The absorbance of samples was measured at 695 nm. The result was expressed as equivalents of ascorbic acid/g of dry sample (μg EAA/g DS) used as a standard.

#### 3.6.3. Size Exclusion Chromatography (SEC)

Gel-exclusion chromatography was performed on Sephadex G-50 gel in order to understand the molecular distribution of phenolic compounds in the six extracts from PR varieties. Tannic acid and catechin were used as references. Briefly, 400 μL of each extract at a concentration of 20 mg/mL was deposited on a Sephadex G-50 column (2 × 40 cm) previously balanced with solvent (NaOH 5 mM and LiCl 2.5 mM) to measure the absorbance at 380 nm by a Shimadzu UV-1600 PC UV spectrophotometer [1] using fractions of 4 mL.

#### 3.6.4. High-Performance Liquid Chromatography with a Diode-Array Detector (HPLC-DAD)

Chromatographic separations of the six pomegranate extracts were performed using an L-3000 high-performance liquid chromatography system (Rigol Technologies, Beijing, China) on a Kinetex EVO C18 column (150 × 4.6 mm, particle size of 5 μm) with a 10 µL injection volume. The mobile phase used was (A) 0.1% trifluoroacetic acid (TFA) in water and (B) 0.1% trifluoroacetic acid (TFA) in acetonitrile at a flow rate of 1 mL/min. The elution gradient used was as follows: 98% A for 10 min, 98–75% A for 25 min, 75–0% A for 20 min, and 0–98% A for 5 min. The column temperature was kept at 30 °C and the detection was monitored at 250, 280, 300, 370, and 510 nm for the identification and quantification of sixteen standard compounds such as tannic acid, gallic acid, (+) catechin, (−) epicatechin, caffeic acid, chlorogenic acid, *p*-coumaric acid, rutin, naringenin, naringin, quercetin, daidzein, genistein, hyperoside, delphinidin, and malvidin, belonging to the pomegranate polyphenolic compound class. Therefore, 250 nm was used for tannic acid, gallic acid, catechin, and rutin, 280 nm was used for chlorogenic acid, and 300 nm was used for caffeic acid. Identification and quantification analyses were carried out by comparison with standard spectra at each retention time. Therefore, 250 nm was used for tannic acid, gallic acid, (+) catechin, rutin, daidzein, and genistein, 280 nm was used for chlorogenic acid, (−) epicatechin, naringenin, and naringin, 300 nm was used for caffeic acid and p-coumaric acid, 370 nm for hyperoside and quercetin, and 510 nm for delphinidin and malvidin. Five data points of concentration between 10 and 400 μg/mL of standard compounds were used for the calibration curve. For the calibration curve of each standard polyphenolic compound, R^2^ was between 0.998 and 0.999, LOD was ±10 μg/mL, and LOQ was equal to or higher than 10 μg/mL [69].

#### 3.6.5. Statistical Analysis

All experiments were performed in triplicate per treatment and were repeated twice. Results were expressed as the means ± standard deviation. Data were graphically reported using the OriginLab 8 Software and data analyses of variance and statistical significance were performed using the statistic software SPSS version 26 (IBM Corp., Armonk, NY, USA). Analysis of variance (ANOVA) was applied to the data. Means corresponding to each variety were compared using Duncan’s multiple range test (*p* < 0.05). Principal component analysis (PCA) was carried out using the SPSS 26 for variables (L: length; W: width; D: diameter; Wg: weight; R: rinds%; J: juice%; S: seed%; Rth: rind thickness; Sratio: seed/weight; Cint: color intern; Cext: color extern; TS: total sugars; TP: total proteins; TA: total acidity; TSS: total soluble solids; MI: maturity index; AA: ascorbic acid content; TPC: total phenolics content; TF: total flavonoids content; TT: total tannins; HT: hydrolyzable tannins content; TCA: total carotenoids content). The PCA score plot was used as a visualization method.

## 4. Conclusions

This work focuses on understanding the particularity of six pomegranate varieties selected with a high commercial interest in the Moroccan market. Local cultivars showed diverse profiles proving their added value either for fresh consumption (L2), juice processing (L4), or rinds valorization (L1, L2) through industrial transformations. On the other hand, imported varieties could be valorized, concurrently with fresh consumption, for juice processing (I2), or rinds valorization (I1). The phenolics contents and antioxidant activity for PJ and PR varieties, to a significant extent, were impacted by the cultivar type. SEC confirmed the high molecular weight of PR extracts and HPLC-DAD showed the abundance of (+) catechin in these extracts. In this context, the comparison of catechin contents of PR varieties allowed the classification of the studied cultivars into three groups: L1- L4, L2-L3, and I1-I2, based on their hierarchy. However, the PCA allowed a specific differentiation pattern in which five groups were distinguished depending on their pomology, juice organoleptic, and physico-chemical properties: I3-I1, I2, L2, L1, and L4. The obtained results provide data information that encourages selecting precisely desirable and highly demanded varieties, especially local ones, which are characterized by a high juice content as a fresh fruit and a catechin-rich source as a vegetable biomass. The groups’ classification obtained will be exploited in further research works for environmental applications

The obtained results provide much information that encourages us to select precisely desirable and highly demanded varieties, especially local ones, which are characterized by high juice content as a fresh fruit and a catechin-rich source as a vegetable biomass.

## Figures and Tables

**Table 1:** Mean (*n* = 15) values of physical characteristics of analyzed pomegranate cultivars.

Variety	Length (cm)	Width (cm)	Diameter (cm)	Weight (g)	Rinds (%)	Juice (%)	Seeds (%)	Rind Thickness (mm)
**L1**	6.41 ± 0.36 ^c^	7.70 ± 0.61 ^d^	8.06 ± 0.50 ^d^	325.33 ± 57.51 ^d^	43.64 ± 6.02 ^c^	52.23 ± 5.58 ^c^	4.12 ± 5.58 ^d^	4.33 ± 0.58 ^c^
**L2**	5.33 ± 0.25 ^a^	6.16 ± 0.35 ^a^	6.20 ± 0.14 ^a^	139.40 ± 11.61 ^a^	43.99 ± 7.71 ^c^	53.85 ± 7.48 ^c^	2.15 ± 0.45 ^a^	3.00 ± 0.00 ^b^
**L3**	7.13 ± 0.21 ^d^	8.50 ± 0.37 ^e^	8.98 ± 0.54 ^e^	479.46 ± 77.16 ^e^	36.95 ± 1.95 ^b^	48.07 ± 1.34 ^b^	3.16 ± 0.14 ^c^	4.90 ± 0.49 ^d^
**L4**	6.4 ± 0.34 ^c^	7.27 ± 0.25 ^c^	7.45 ± 0.28 ^c^	271.06 ± 31.36 ^c^	28.33 ± 3.94 ^a^	67.11 ± 4.12 ^e^	4.55 ± 0.36 ^e^	2.60 ± 0.53 ^a^
**I1**	7.22 ± 0.26 ^d^	8.31 ± 0.21 ^e^	8.67 ± 0.31 ^e^	447.26 ± 53.82 ^e^	45.27 ± 1.64 ^c^	12.34 ± 1.34 ^a^	2.77 ± 0.33 ^b^	5.46 ± 0.57 ^e^
**I2**	6.08 ± 0.43 ^b^	6.99 ± 0.20 ^b^	6.96 ±0.16 ^b^	203.20 ± 14.20 ^b^	37.48 ± 4.03 ^b^	59.54 ± 4.23 ^d^	2.96 ±0.39 ^bc^	2.83 ± 0.25 ^ab^

The values followed by the same letter a, b, c, d, e in the same column show no statistically significant differences (*p* < 0.05). Mean values (*n* = 3) ± standard error.

**Table 2:** Mean (*n* = 15) values of seeds and arils physical characteristics.

Variety	Arils Weight (g)	Seeds Weight (g)	Seed Length (mm)	Seed Width (mm)	Seed Weight/Aril Weight (%)
**L1**	180 ± 26.97 ^c^	13.16 ± 3.12 ^c^	6.11 ± 0.30 ^a^	2.58 ± 0.33 ^c^	7.25 ± 0.66 ^d^
**L2**	80.12 ± 14.49 ^a^	3.14 ± 0.87 ^a^	6.45 ± 0.23 ^b^	2.13 ± 0.13 ^a^	3.90 ± 0.71 ^a^
**L3**	302.16 ± 44.50 ^e^	15.33 ± 2.33 ^d^	6.38 ± 0.54 ^ab^	2.33 ± 0.23 ^ab^	5.08 ± 0.36 ^b^
**L4**	196.14 ± 28.31 ^c^	12.28 ± 0.95 ^c^	6.58 ± 0.44 ^b^	2.34 ± 0.13 ^b^	6.34 ± 0.75 ^c^
**I1**	243.75 ± 33.68 ^d^	12.29 ± 2.68 ^c^	6.37 ± 0.59 ^ab^	2.7 ± 0.24 ^c^	5.02 ± 0.67 ^b^
**I2**	128.16 ± 14.17 ^b^	6 ± 0.63 ^b^	7.15 ± 0.53 ^c^	2.66 ± 0.25 ^c^	4.75 ± 0.83 ^b^

The values followed by the same letter a, b, c, d, e in the same column show no statistically significant differences (*p* < 0.05). Mean values (*n* = 3) ± standard error.

**Table 3:** Some organoleptic properties of PJ varieties.

Variety	Seediness	Fruit Flavor			Color Intern	Color Extern	Red Color
		Sweetness	Acidity	Astringency			
**L1**	3	2	1	3	2	1	1
**L2**	1	2	2	2	1	4	2
**L3**	2	2	1	3	2	3	1
**L4**	3	2	2	2	1	2	2
**I1**	2	3	1	1	3	4	2
**I2**	2	1	2	1	4	5	3

Encoding: Seediness: soft-seeded = 1; semi-seeded = 2; hard-seeded = 3; Sweetness: low = 1; medium = 2; high = 3; Acidity: low = 1; medium = 2; high = 3; Astringency: low = 1; medium = 2; high = 3; Color intern: dark pink = 1; light red = 2; red = 3; dark red = 4; Color extern: yellow = 1; yellow-orange = 2; orange = 3; orange-red = 4; dark red = 5; Red color: light = 1; medium = 2; dark = 3.

**Table 4:** Mean values of some chemical characteristics of PJ varieties.

Variety	TS (g/100 mL)	TP (g/100 mL)	pH	TA (g Citric acid/L)	TSS (Brix°)	MI (%)	Description
**L1**	13.10 ± 0.01 ^a^	1.16 ± 0.02 ^c^	3.67 ± 0.06 ^a^	2.9 ± 0.07 ^b^	20.9 ± 0.06 ^e^	71.91 ± 1.97 ^d^	Sweet
**L2**	12.93 ± 0.04 ^a^	1.01 ± 0.004 ^b^	4.37 ± 0.05 ^c^	4.9 ± 0.07 ^c^	18.3 ± 0.06 ^b^	37.35 ± 0.62 ^b^	Sweet
**L3**	11.17 ± 0.01 ^a^	0.66 ± 0.09 ^a^	4.35 ± 0.05 ^c^	2.6 ±0.20 ^a^	20.2 ± 0.05 ^d^	77.66 ± 5.83 ^e^	Sweet
**L4**	12.99 ± 0.02 ^a^	0.95 ± 0.02 ^b^	3.85 ± 0.05 ^b^	2.8 ± 0.13 ^ab^	19.4 ± 0.10 ^c^	68.94 ± 2.96 ^d^	Sweet
**I1**	13.18 ± 0.008 ^a^	1.03 ± 0.15 ^b^	3.89 ± 0.07 ^b^	4.9 ± 0.07 ^c^	29.2 ± 0.06 ^f^	59.41 ± 0.84 ^c^	Sweet
**I2**	12.83 ± 0.04 ^a^	1.01 ± 0.05 ^b^	4.56 ± 0.06 ^d^	5.5 ± 0.15 ^d^	12.8 ± 0.06 ^a^	22.96 ± 0.66 ^a^	Sour-sweet

TS: total sugars; TP: total proteins; TA: total acidity; TSS: total soluble solids; MI: maturity index. The values followed by the same letter a, b, c, d, e, f in the same column show no statistically significant differences (*p* < 0.05). Mean values (*n* = 3) ± standard error.

**Table 5:** Vitamin C, phenolic compounds, and total carotenoids contents of PJ varieties.

Variety	AA (mg AAE/L)	TPC (g GAE/L)	TF (mgQE/100 mL)	TT (mg/100 mL)	HT (mgTAE/100 mL)	TCA (mg β-CAE/100 mL)
**L1**	842.76 ± 0.05 ^b^	1.222 ± 0.211 ^b^	47.07 ± 1.46 ^b^	385.79 ± 5.03 ^b^	272.92 ± 4.72 ^d^	0.12 ± 0.001 ^a^
**L2**	851.56 ± 0.05 ^c^	1.090 ± 0.051 ^ab^	59.42 ± 0.41 ^d^	294.95 ± 5.56 ^a^	192.12 ± 4.16 ^b^	1.02 ± 0.003 ^d^
**L3**	833.96 ± 0.05 ^a^	0.885 ± 0.106 ^a^	62.47 ± 2.45 ^e^	966.50 ± 5.99 ^e^	314.39 ± 5.50 ^e^	0.65 ± 0.01 ^c^
**L4**	845.70 ± 0.05 ^b^	1.216 ± 0.210 ^b^	41.66 ± 2.36 ^a^	713.81 ± 6.50 ^d^	160.96 ± 7 ^a^	1.53 ± 0.001 ^e^
**I1**	831.03 ± 0.05 ^a^	0.995 ± 0.265 ^a^	55.53 ± 2.49 ^c^	1456.14 ± 11.06 ^f^	241.97 ± 8.73 ^c^	1.87 ± 0.01 ^f^
**I2**	831.03 ± 0.05 ^a^	1.348 ± 0.05 ^b^	140.44 ± 1.80 ^f^	634.89 ± 8.32 ^c^	362.51 ± 5.29 ^f^	0.48 ± 0.03 ^b^

AA: ascorbic acid content; TPC: total phenolics content; TF: total flavonoids content; TT: total tannins; HT: hydrolyzable tannins content; TCA: total carotenoids content. The results were expressed based on fresh PJ. The values followed by the same letter a, b, c, d, e, f in the same column show no statistically significant differences (*p* < 0.01). Mean values (*n* = 3) ± standard error.

**Table 6:** Loadings, eigenvalues, and percent of cumulative variance for the first two principal components.

Variable	Component	
	1 38.17%	2 64.53%
**L**	0.937	0.201
**W**	0.950	0.165
**D**	0.967	0.111
**Wg**	0.976	0.171
**R**	0.072	0.384
**J**	−0.615	−0.508
**S**	0.257	−0.748
**Rth**	0.863	0.319
**S ratio**	0.359	−0.703
**C int**	0.011	0.774
**C ext**	−0.379	0.872
**TS**	−0.373	−0.222
**TP**	−0.363	−0.230
**pH**	−0.501	0.663
**TA**	−0.529	0.705
**TSS**	0.776	0.002
**MI**	0.821	−0.534
**AA**	−0.448	−0.733
**TPC**	−0.690	−0.208
**TF**	−0.519	0.687
**TT**	0.741	0.480
**HT**	0.029	0.650
**TCA**	0.269	0.065

L: length; W: width; D: diameter; Wg: weight; R: rinds%; J: juice%; S: seed%; Rth: rind thickness; Sratio: seed/weight; Cint: color intern; Cext: color extern; TS: total sugars; TP: total proteins; TA: total acidity; TSS: total soluble solids; MI: maturity index; AA: ascorbic acid content; TPC: total phenolics content; TF: total flavonoids content; TT: total tannins; HT: hydrolysable tannins content; TCA: total carotenoids content.

**Table 7:** Correlation matrix between phenolics contents and antioxidant activity of pomegranate varieties rinds.

	1	2	3	4	5	6
**1. TPC**	1					
**2. TFC**	0.71	1				
**3. CTC**	−0.40	−0.59	1			
**4. DPPH** **^•^**	−0.72 *	−0.95 **	**0.63**	1		
**5. ABTS** **^•+^**	−0.14	−0.54	0.89 *	0.46	1	
**6. TAC**	**0.62**	−0.05	−0.10	0.022	0.14	1

TPC: total phenolic compounds; TFC: total flavonoids content; CTC: condensed tannins content; DPPH^•^: IC_50_ (DPPH^•^) obtained at 15 min of incubation; ABTS^•+^: IC_50_ (ABTS^•+^) obtained at 15 min of incubation, TAC: total antioxidant capacity; ** *p* ≤ 0.01; * *p* ≤ 0.05.

**Table 8:** Quantification data of identified PR varieties phenolic compounds by λmax each standard compound by HPLC-DAD analysis.

Compound Name	T_R_ (min)	Concentration (mg/kg dw)					
		L1	L2	L3	L4	I1	I2
**Tannic acid**	1.90	0.35	0.26	0.54	0.46	0.39	0.38
**Gallic acid**	3.77	0.58	0.39	ND	ND	ND	0.63
**(+) Catechin**	19.60	690.84	115.79	93.63	1579.91	486.88	403.65
**Caffeic acid**	20.25	0.10	0.12	0.23	0.27	0.20	0.19
**Chlorogenic acid**	20.93	1.74	0.52	0.96	1.19	1.91	1.20
**Rutin**	29.10	2.41	1.11	3.19	4.29	3.28	1.08

ND: not determined. Retention time (T_R_) error of mean for compounds was ± 0.0001–0.2 min. Data were expressed as mg/kg on a dry weight basis.

**Table 9:** Origins geographic of the six pomegranate commercial cultivars collected.

	Code	Variety Name	Origins Geographic
Local	L1	Djeibi	Morocco
	L2	Mersi	
	L3	Sefri 1	
	L4	Sefri 2	
Imported	I1	Mollar de Elche	Spain
	I2	Hicaz	Turkey

## Data Availability

Not applicable.
